# Impact of Cattaneo-Christov heat flux model on MHD hybrid nano-micropolar fluid flow and heat transfer with viscous and joule dissipation effects

**DOI:** 10.1038/s41598-020-77419-x

**Published:** 2021-01-11

**Authors:** Asifa Tassaddiq

**Affiliations:** Department of Basic Sciences and Humanities, College of Computer and Information Sciences, Majmaah University, Al-Majmaah, 11952 Saudi Arabia

**Keywords:** Materials science, Mathematics and computing

## Abstract

Review of literature reveals that hybrid nanofluids are more effective for heat transmission as compared to the conventional fluids. Nevertheless, the knowledge of developed techniques for the enhancement of heat transmission in hybrid nanofluids has many gaps and, subsequently, an extensive study for such fluids is vital. In this article, the author investigates the effect of hybrid nanoparticles on the thermal efficiency of nano-structured nanoparticles (micropolar fluid) by using the Cattaneo-Christov heat flux model. The magnetic field is pragmatic normal to the hybrid nanofluid flow direction. In order to investigate the influence of physical parameters, the proposed model has been converted to a set of ordinary differential equations (ODEs) by means of involved variables. Furthermore, the analytical and numerical approaches are compared by using different techniques to comprehend the significance of this research. It is found that macro-velocity field reduces with micropolar factor and Hartmann number. A significant result is found in micro-velocity field for the cases when $n = 0.5$ and $n = 0$. Also an escalating conduct in thermal field is observed against the increasing estimations of Hartmann number, micropolar parameter, Eckert number, and material parameter.

## Introduction

Enhancing the thermal transfer of operating nanoparticles by dispersing them has drawn as much scientific publicity as is evidenced by the fact that the distribution of nanomaterials enhances the operative thermal conductivity of these nanomaterials in which these nanoparticles are distributed. Theoretical and experimental research shows that functioning isolated fluids have comparatively poor thermal transfer capabilities compared to nanoparticle-containing fluids. Spectacular increase in the thermal efficiency of combinations consisting of nano-sized metallic particles has now been identified. After Choi's groundbreaking work^1^, several scientists have performed experimental and theoretical analyses from diverse characteristics concerning the benefits of nanoparticles in thermal scheme. Nonetheless, some of the most recent and interconnected analysis have been identified. For instance, Dogonchi et al.^2^ analyzed the function for the distribution of Fe_3_O_4_ in strengthening thermal transfer in the jar. They observed the consequences of thermal energy and the function of nanoparticles in the production of drugs throughout chemotherapy. Dogonchi and Hashim^3^ deliberated the influence of Fe_3_O_4_ on the flow of water in a round tube. They even spoke about flow in a jar like such a rhombus. Various studies were implemented to examine the effect of magnetic dipole-dependent viscosity on just the transportation of energy in a nanofluid. Sheikholeslami et al.^4^ formulated the transmission of phenomena in the porous medium subject to the magnetic field and developed computational formulations to explore the effect of nanoparticles on the energy transmission in water. Sheikholeslami and Rokni^5^ explored the effect of the Coulomb force on the energy transportation in nanofluid through the cavity. Safaei et al.^6^ have established computational models for the operation of hybrid nanomaterials in heat transmission to a permeable media that are subjected to imposed magnetic and have resolved formulated challenges to investigate the consequences of the resulting factors on the heat transmission. Safaei et al.^7^ assessed the effect of ZnO-TiO on ethylene glycol thermal conductivity. Nadeem et al.^8^ evaluated the effect of free-flow momentum on conduction in a water mixture containing Cu and Al_2_O_3_ under thermal slip procedure. Saleem et al.^9^ carried out the study of the effect of thermal origin and viscous dissipation in nano-material released heat radiation. Sadiq et al.^10^ conducted the simulations for the movement of the magnetic field linked through micropolar fluid and the nanoparticles dispersion. Other interrelated studies were published in^11–16^. The incompressible flow of hybrid dusty fluid using Darcian medium with Cattaneo-Christov model was discussed by Ganesh et al.^17^. In another article Reddy et al.^18^ investigated the two-phase flow of Oldroyd-B fluid using Cattaneo-Christov model. Shah et al.^19–21^ recently investigated the effects of heat transfer and thermal radiation on nanofluid flow in different geometries. Heat transfer features are studied with details in their work. Analytical and numerical approached are applied for the best solution of obtained modelled.

The flow of micropolar fluid initiated by Eringin^22^ is concentrating presently. Ferrofluid, liquid crystals, blood, suspensions etc. are examples of micropolar fluid. The micropolar fluid theory presented by Eringin is built on continuum hypothesis. This theory states that those fluids which are composed of randomly oriented, rigid, or spherical particles with their specific microrotations or spins are known as micropolar fluids. The stagnation point micropolar fluid flow was determined by Guram and smith^23^. The micropolar fluid flow over axisymmetric sheets with heat source was investigated by Gorla and Takhar^24^. The stagnation flow of micropolar fluid with combine convective conditions was presented by Gorla et al.^25^. Sedeek^26^ probed the micropolar fluid flow past a moving sheet with magnetic field influence by means of micropolar fluid theory. Nazar et al.^27^ determined the stagnation point of a micropolar viscous fluid flow. The micropolar fluid flow and heat transmission by means of finite element technique was discussed by Takhar et al.^28^. The heat transmission in a micropolar fluid flow in a porous media was addressed by Eldahab et al.^29^. Nazar et al.^30^ scrutinized the boundary layer flow of micropolar fluid on a sphere. For more understanding and analysis of the relevant nano fluid flow, the interested reader is referred to recent researches^31–39^.

By means of a systematic review, the researchers have found that the analysis (using HAM and Shooting) on the consequence of hybrid nanoparticles on the thermal efficiency of nano-structured nanoparticles (micropolar fluid) has not yet been performed. The Cattaneo-Christov heat flux model is performed in the current investigation. This research will fill this space in current scientific innovations. The present research problem is solved analytically and numerically as well. The results obtained are presented graphically and discussed briefly.

## Problem formulation

**Figure 1:**
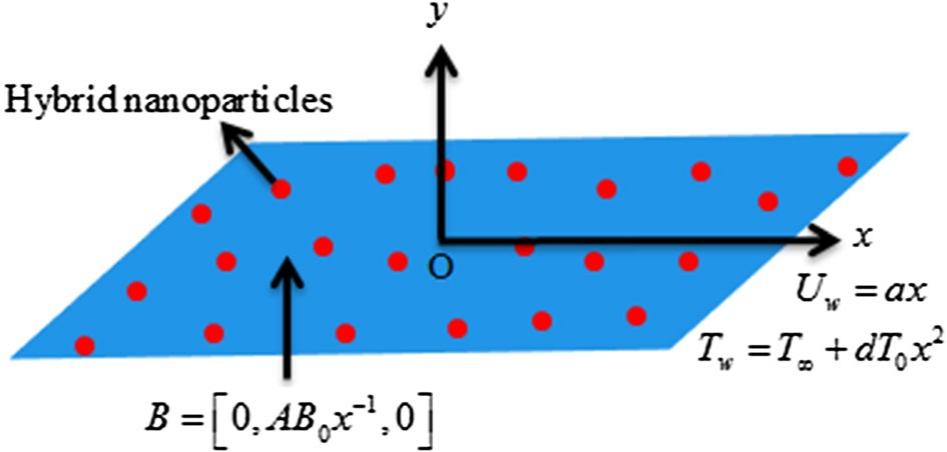
Fluid flow geometry.

Here it is assumed the micropolar fluid flow over an elastic body with Cattaneo-Christov heat flux model conditional on dispersion of hybrid nanoparticles ${\text{Cu}}$ and ${\text{Al}}_{2} {\text{O}}_{3}$. The elastic body is moving with velocity $U_{w} \left( x \right) = ax$. The temperature of the stretchable body is taken as $T_{w} \left( x \right) > T$ where $T$ and $T_{w}$ is the fluid’s temperature and the stretchable surface’s temperature of the elastic body. The magnetic field $B = \left[ {0,\,AB_{0} x^{ - 1} ,\,0} \right]$ is practiced vertical to the fluid flow as shown in Fig. 1.

1 $$ \frac{\partial u}{{\partial x}} + \frac{\partial v}{{\partial y}} = 0, $$

2 $$ u\frac{\partial u}{{\partial x}} + v\frac{\partial u}{{\partial y}} = \frac{1}{{\rho_{hnf} }}\left( {\mu_{hnf} + \lambda_{hnf} } \right)\frac{{\partial^{2} u}}{{\partial y^{2} }} - \frac{{\sigma_{hnf} A^{2} B_{0}^{2} x^{ - 2} }}{{\rho_{hnf} }}u - \frac{{\lambda_{hnf} }}{{\rho_{hnf} }}\frac{\partial N}{{\partial y}}, $$

3 $$ \rho_{hnf} j\left( {u\frac{\partial N}{{\partial x}} + v\frac{\partial N}{{\partial y}}} \right) = \gamma_{hnf} \frac{{\partial^{2} N}}{{\partial y^{2} }} - \lambda_{hnf} \left( {2N + \frac{\partial u}{{\partial y}}} \right), $$

4 $$ \begin{aligned} \left( {\rho c_{p} } \right)_{hnf} \left( {u\frac{\partial T}{{\partial x}} + v\frac{\partial T}{{\partial y}}} \right) & = k_{hnf} \frac{{\partial^{2} T}}{{\partial y^{2} }} + \sigma_{hnf} A^{2} B_{0}^{2} x^{ - 2} u^{2} + \left( {\mu_{hnf} + \frac{{\lambda_{hnf} }}{2}} \right)\left( {\frac{\partial u}{{\partial y}}} \right)^{2} \hfill \\  & \quad - \lambda_{1} \left( \begin{gathered} u\frac{\partial T}{{\partial x}}\frac{\partial u}{{\partial x}} + u\frac{\partial T}{{\partial y}}\frac{\partial v}{{\partial x}} + v\frac{\partial T}{{\partial y}}\frac{\partial v}{{\partial y}} + v\frac{\partial T}{{\partial x}}\frac{\partial u}{{\partial y}} \hfill \\ + 2vu\frac{{\partial^{2} T}}{\partial x\partial y} + u^{2} \frac{{\partial^{2} T}}{{\partial x^{2} }} + v^{2} \frac{{\partial^{2} T}}{{\partial y^{2} }} \hfill \\ \end{gathered} \right) \\ & \quad + \frac{{\lambda_{hnf} }}{2}\left( {\frac{\partial u}{{\partial y}} - 2N} \right)^{2} + \left( {\alpha + \beta + \gamma } \right)\left( {\frac{\partial N}{{\partial y}}} \right)^{2} , \hfill \\ \end{aligned} $$

With

5 $$ \begin{gathered} u = U_{w} x,\,\,v = 0,\,\,T = T_{w} ,\,\,N = - n\frac{\partial u}{{\partial y}}\,\,\,{\text{at}}\,\,\,y = 0, \hfill \\ u \to 0,\,\,\,T \to T_{\infty } ,\,\,\,N \to 0\,\,\,as\,\,\,y \to \infty . \hfill \\ \end{gathered} $$

Here $n$ represents the constant lies in $\left[ {0\,\,\,1} \right]$. For the situation when micro-motion disappears at the surface i.e. $n = 0$. For the situation when micro-motion not disappears at the surface, the constant i.e. $n = 0.5$, and $n = 1$ is for the situation of turbulent flow.

The thermo-physical properties used in the modeled problem are defined by

6 $$ \begin{aligned} \frac{{\rho_{hnf} }}{{\rho_{f} }} & = \left( {1 - \Phi_{2} } \right)\left\{ {1 - \Phi_{1} + \Phi_{1} \frac{{\rho_{S1} }}{{\rho_{f} }}} \right\} + \Phi_{2} \frac{{\rho_{S2} }}{{\rho_{f} }},\,\,\mu_{hnf} = \frac{{\mu_{f} }}{{\left( {1 - \Phi_{1} } \right)^{{{5 \mathord{\left/ {\vphantom {5 2}} \right. \kern-\nulldelimiterspace} 2}}} \left( {1 - \Phi_{2} } \right)^{{{5 \mathord{\left/ {\vphantom {5 2}} \right. \kern-\nulldelimiterspace} 2}}} }}, \hfill \\ \frac{{\left( {\rho c_{p} } \right)_{hnf} }}{{\left( {\rho c_{p} } \right)_{f} }} & = \left( {1 - \Phi_{2} } \right)\left\{ {1 - \Phi_{1} + \Phi_{1} \frac{{\left( {\rho c_{p} } \right)_{S1} }}{{\left( {\rho c_{p} } \right)_{f} }}} \right\} + \Phi_{2} \frac{{\left( {\rho c_{p} } \right)_{S2} }}{{\left( {\rho c_{p} } \right)_{f} }},\,\,\,\lambda_{hnf} = \frac{{\lambda_{f} }}{{\left( {1 - \Phi_{1} } \right)^{{{5 \mathord{\left/ {\vphantom {5 2}} \right. \kern-\nulldelimiterspace} 2}}} \left( {1 - \Phi_{2} } \right)^{{{5 \mathord{\left/ {\vphantom {5 2}} \right. \kern-\nulldelimiterspace} 2}}} }}, \hfill \\ \frac{{k_{hnf} }}{{k_{bf} }} & = \frac{{2k_{bf} + k_{S2} - 2\Phi_{2} \left( {k_{bf} - k_{S2} } \right)}}{{2k_{bf} + k_{S2} + \Phi_{2} \left( {k_{bf} - k_{S2} } \right)}},\,\,\,\frac{{k_{bf} }}{{k_{f} }} = \frac{{2k_{f} + k_{S1} - 2\Phi_{1} \left( {k_{f} - k_{S1} } \right)}}{{2k_{f} + k_{S1} + \Phi_{1} \left( {k_{f} - k_{S1} } \right)}}, \hfill \\ \frac{{\sigma_{hnf} }}{{\sigma_{bf} }} & = \frac{{2\sigma_{bf} + \sigma_{S2} - 2\Phi_{2} \left( {\sigma_{bf} - \sigma_{S2} } \right)}}{{2\sigma_{bf} + \sigma_{S2} + \Phi_{2} \left( {\sigma_{bf} - \sigma_{S2} } \right)}},\,\,\,\frac{{\sigma_{bf} }}{{\sigma_{f} }} = \frac{{2\sigma_{f} + \sigma_{S1} - 2\Phi_{1} \left( {\sigma_{f} - \sigma_{S1} } \right)}}{{2\sigma_{f} + \sigma_{S1} + \Phi_{1} \left( {\sigma_{f} - \sigma_{S1} } \right)}}. \hfill \\ \end{aligned} $$

Here, the subscripts $S1\,\,{\text{and}}\,\,S2$ are used for ${\text{Cu}}$ and ${\text{Al}}_{2} {\text{O}}_{3}$ nanoparticles respectively. The nano-particles’ thermo-physical properties and micro polar fluid are displayed in Table 1.

**Table 1:** The nano-particles’ thermo-physical properties and micro polar fluid.

Physical property	${\text{Cu}}$	${\text{Al}}_{2} {\text{O}}_{3}$	Blood
${\rho \mathord{\left/ {\vphantom {\rho {\left( {{\text{kg}}\;{\text{m}}^{ - 3} } \right)}}} \right. \kern-\nulldelimiterspace} {\left( {{\text{kg}}\;{\text{m}}^{ - 3} } \right)}}$	8933	3970	1060
${{c_{p} } \mathord{\left/ {\vphantom {{c_{p} } {\left( {{\text{J}}\;{\text{kg}}^{ - 1} {\text{K}}^{ - 1} } \right)}}} \right. \kern-\nulldelimiterspace} {\left( {{\text{J}}\;{\text{kg}}^{ - 1} {\text{K}}^{ - 1} } \right)}}$	385	765	3770
${k \mathord{\left/ {\vphantom {k {\left( {{\text{W}}\;{\text{m}}^{ - 1} {\text{K}}^{ - 1} } \right)}}} \right. \kern-\nulldelimiterspace} {\left( {{\text{W}}\;{\text{m}}^{ - 1} {\text{K}}^{ - 1} } \right)}}$	401	40	40
$\Phi$	0.05	0.15	0.0
${\sigma \mathord{\left/ {\vphantom {\sigma {\left( {{\text{s}}\;{\text{m}}^{ - 1} } \right)}}} \right. \kern-\nulldelimiterspace} {\left( {{\text{s}}\;{\text{m}}^{ - 1} } \right)}}$	59.6 × 10^6^	35 × 10^6^	4.3 × 10^–5^

The similarity variables are defined by

7 $$ \begin{aligned} u & = \frac{\partial \psi }{{\partial y}},\,\,v = - \frac{\partial \psi }{{\partial x}},\,\,\,\psi \left( {x,y} \right) = \left( {a\upsilon_{f} } \right)^{{{1 \mathord{\left/ {\vphantom {1 2}} \right. \kern-\nulldelimiterspace} 2}}} xf\left( \xi \right), \hfill \\ \theta \left( \xi \right) & = \frac{{T - T_{\infty } }}{{T_{w} - T_{\infty } }},\,\,\,N\left( \xi \right) = x\left( {\frac{{a^{3} }}{{\upsilon_{f} }}} \right)^{{{1 \mathord{\left/ {\vphantom {1 2}} \right. \kern-\nulldelimiterspace} 2}}} g\left( \xi \right),\,\,\,\xi = \left( {\frac{a}{{\upsilon_{f} }}} \right)^{{{1 \mathord{\left/ {\vphantom {1 2}} \right. \kern-\nulldelimiterspace} 2}}} y. \hfill \\ \end{aligned} $$

On substituting (7) in (2–5), we have

8 $$ \begin{gathered} \left( {1 + K} \right)f^{\prime\prime\prime} + Kg^{\prime} + \frac{{\sigma_{hnf} }}{{\sigma_{f} \left[ {\left( {1 - \Phi_{2} } \right)\left\{ {1 - \Phi_{1} + \Phi_{1} \frac{{\rho_{S1} }}{{\rho_{f} }}} \right\} + \Phi_{2} \frac{{\rho_{S2} }}{{\rho_{f} }}} \right]}}Haf^{\prime} \hfill \\ - \left( {1 - \Phi_{1} } \right)^{2.5} \left( {1 - \Phi_{2} } \right)^{2.5} \left[ {\left( {1 - \Phi_{2} } \right)\left\{ {1 - \Phi_{1} + \Phi_{1} \frac{{\rho_{S1} }}{{\rho_{f} }}} \right\} + \Phi_{2} \frac{{\rho_{S2} }}{{\rho_{f} }}} \right]\left( {f^{{\prime}{2}} - ff^{\prime\prime}} \right) = 0, \hfill \\ \end{gathered} $$

9 $$ \left( {1 + \frac{K}{2}} \right)g^{\prime\prime} - K\left( {2g + f^{\prime\prime}} \right) - \left( {1 - \Phi_{1} } \right)^{2.5} \left( {1 - \Phi_{2} } \right)^{2.5} \left[ {\left( {1 - \Phi_{2} } \right)\left\{ \begin{gathered} 1 - \Phi_{1} + \hfill \\ \Phi_{1} \frac{{\rho_{S1} }}{{\rho_{f} }} \hfill \\ \end{gathered} \right\} + \Phi_{2} \frac{{\rho_{S2} }}{{\rho_{f} }}} \right]\left( {f^{\prime}g - fg^{\prime}} \right) = 0, $$

10 $$ \begin{gathered} \frac{{k_{hnf} }}{{k_{f} }}\theta^{\prime\prime} + \frac{{\sigma_{hnf} }}{{\sigma_{f} }}Ha\Pr Ecf^{{\prime}{2}} + \left( {1 + \frac{K}{2}} \right)\frac{Ec\Pr }{{\left( {1 - \Phi_{1} } \right)^{2.5} \left( {1 - \Phi_{2} } \right)^{2.5} }}f^{{\prime\prime}{2}} \hfill \\ + \frac{K}{2}\frac{Ec\Pr }{{\left( {1 - \Phi_{1} } \right)^{2.5} \left( {1 - \Phi_{2} } \right)^{2.5} }}\left( {f^{{\prime\prime}{2}} + 4g^{2} - 4gf^{\prime\prime}} \right) + CEc\Pr {\text{Re}} g^{{\prime}{2}} - \kappa \left( {ff^{\prime}\theta^{\prime} + f^{2} \theta^{\prime\prime}} \right) \hfill \\ + \Pr \left[ {\left( {1 - \Phi_{2} } \right)\left\{ {1 - \Phi_{1} + \Phi_{1} \frac{{\left( {\rho c_{p} } \right)_{S1} }}{{\left( {\rho c_{p} } \right)_{f} }}} \right\} + \Phi_{2} \frac{{\left( {\rho c_{p} } \right)_{S2} }}{{\left( {\rho c_{p} } \right)_{f} }}} \right]\left( {f\theta^{\prime} - f^{\prime}\theta } \right) = 0, \hfill \\ \end{gathered} $$

11 $$ \begin{aligned} f & = 0,\,f^{\prime} = 1,g^{\prime} = - nf^{\prime\prime},\,\theta = 1,\,\,\,\,{\text{at}}\,\,\,\xi = 0\,\, \hfill \\ f^{\prime} & = 0,\,\,g^{\prime} = 0,\,\theta = 0\,\,\,\,{\text{as}}\,\,\,\xi \to \infty . \hfill \\ \end{aligned} $$

The dimensionless variables are defined as

12 $$ \begin{aligned} Ha & = \frac{{\sigma_{f} A^{2} B_{0}^{2} x^{2} }}{{a\rho_{f} }},\,\,\,K = \frac{{\lambda_{f} }}{{\mu_{f} }},\,\,\,\Pr = \frac{{\mu_{f} \left( {c\rho_{p} } \right)_{f} }}{{k_{f} }},\,\,\,\kappa = \lambda a, \hfill \\ Ec & = \frac{{a^{2} x^{2} }}{{\left( {T_{w} - T_{\infty } } \right)\left( {c_{p} } \right)_{f} }},\,\,\,{\text{Re}} = \frac{{ax^{2} }}{{\upsilon_{f} }},\,\,\,C = \frac{{\left( {\alpha + \beta + \gamma } \right)}}{{x^{2} \mu_{f} }},\,\, \hfill \\ \end{aligned} $$

where $Ha$ indicates the Hartmann number, $K$ represents the micropolar parameter, $\Pr$ signifies the Prandtl number, $Ec$ represents the Eckert number,$\,{\text{Re}}$ indicates the Reynolds number, $C$ represents the material parameter, and $\kappa$ signifies the thermal relaxation parameter.

13 $$ \begin{aligned} C_{f} & = \frac{{1 + \frac{K}{2}}}{{\left( {\text{Re}} \right)^{{{1 \mathord{\left/ {\vphantom {1 2}} \right. \kern-\nulldelimiterspace} 2}}} \left( {1 - \Phi_{1} } \right)^{2.5} \left( {1 - \Phi_{2} } \right)^{2.5} }}f^{\prime\prime}\left( 0 \right), \hfill \\ C_{g} & = \frac{{1 + \frac{K}{2}}}{{\left( {\text{Re}} \right)^{{{1 \mathord{\left/ {\vphantom {1 2}} \right. \kern-\nulldelimiterspace} 2}}} \left( {1 - \Phi_{1} } \right)^{2.5} \left( {1 - \Phi_{2} } \right)^{2.5} }}g^{\prime}\left( 0 \right), \hfill \\ Nu & = - \left( {\text{Re}} \right)^{{{1 \mathord{\left/ {\vphantom {1 2}} \right. \kern-\nulldelimiterspace} 2}}} \frac{{k_{hnf} }}{{k_{f} }}\theta^{\prime}\left( 0 \right). \hfill \\ \end{aligned} $$

## Analytical solution

To proceed the modeled equations by using HAM, we assumed that $f = F,\,\,g = G,\,\,\theta = T\,$ and $\xi = \psi$. In understanding of (8–10) with (11), HAM is used with the following procedure.

*Preliminary assumptions*

14 $$ F_{0} (\psi ) = 1 - e^{ - \psi } ,\,\,G_{0} (\psi ) = - ne^{ - \psi } ,\,\,T_{0} (\psi ) = e^{ - \psi } . $$

*Linear operators*

15 $$ L_{F} \left( F \right) = \left( {F^{\prime\prime\prime} - F^{\prime}} \right),\,\,{\text{L}}_{G} \left( G \right) = \left( {G^{\prime\prime} - G} \right),\,\,{\text{L}}_{T} \left( T \right) = \left( {T^{\prime\prime} - T} \right), $$

with

16 $$ L_{F} \left( {p_{1} + p_{2} e^{ - \psi } + p_{3} e^{\psi } } \right) = 0,{\text{ L}}_{G} \left( {p_{4} e^{ - \psi } + p_{5} e^{\psi } } \right) = 0,{\text{L}}_{T} \left( {p_{6} e^{ - \psi } + p_{7} e^{\psi } } \right) = 0. $$

where $p_{i} (i = 1 - 7)$ are constants in general solution.

*0th-order problems*

Assume that $S \in \left[ {0,\,\,1} \right]$ be the rooted factor and $h_{F}$, $h_{G}$ and $h_{T}$ are the non-zero auxiliary factors. then 0th-order problems are

17 $$ \left( {1 - S} \right)L_{F} \left[ {F\left( {\psi ;S} \right) - F_{0} \left( \psi \right)} \right] = S\hbar_{F} \aleph_{F} \left[ {F\left( {\psi ;S} \right),F\left( {\psi ;S} \right)} \right], $$

18 $$ \left( {1 - S} \right)L_{G} \left[ {G\left( {\psi ;S} \right) - G_{0} \left( \psi \right)} \right] = S\hbar_{G} \aleph_{G} \left[ {G\left( {\psi ;S} \right),F\left( {\psi ;S} \right)} \right], $$

19 $$ \left( {1 - S} \right)L_{T} \left[ {T\left( {\psi ;S} \right) - T_{0} \left( \psi \right)} \right] = S\hbar_{T} \aleph_{T} \left[ {T\left( {\psi ;S} \right),F\left( {\psi ;S} \right),G\left( {\psi ;S} \right)} \right], $$

20 $$ \begin{gathered} F\left( {0;S} \right) = 0,\,\,\,F^{\prime}\left( {0;S} \right) = 1,\,\,G^{\prime}\left( {0;S} \right) = - nF^{\prime\prime}\left( {0;S} \right),\,\,\,T\left( {0;S} \right) = 1,\, \hfill \\ F^{\prime}\left( {\infty ;S} \right) = 0,\,\,\,G^{\prime}\left( {\infty ;S} \right) = 0,\,\,\,T\left( {\infty ;S} \right) = 0\,. \hfill \\ \end{gathered} $$

21 $$ \begin{gathered} \aleph_{F} \left[ {F\left( {\psi ;S} \right),g\left( {\psi ;S} \right)} \right] = \left( {1 + K} \right)\frac{{\partial^{3} F\left( {\psi ;S} \right)}}{{\partial \psi^{3} }} + K\frac{{\partial G\left( {\psi ;S} \right)}}{\partial \psi } \hfill \\ + \frac{{\sigma_{hnf} }}{{\sigma_{f} \left[ {\left( {1 - \Phi_{2} } \right)\left\{ {1 - \Phi_{1} + \Phi_{1} \frac{{\rho_{S1} }}{{\rho_{f} }}} \right\} + \Phi_{2} \frac{{\rho_{S2} }}{{\rho_{f} }}} \right]}}Ha\frac{{\partial F\left( {\psi ;S} \right)}}{\partial \psi } \hfill \\ - \left( {1 - \Phi_{1} } \right)^{2.5} \left( {1 - \Phi_{2} } \right)^{2.5} \left[ {\left( {1 - \Phi_{2} } \right)\left\{ \begin{gathered} 1 - \Phi_{1} \hfill \\ + \Phi_{1} \frac{{\rho_{S1} }}{{\rho_{f} }} \hfill \\ \end{gathered} \right\} + \Phi_{2} \frac{{\rho_{S2} }}{{\rho_{f} }}} \right]\left( \begin{gathered} \left( {\frac{{\partial F\left( {\psi ;S} \right)}}{\partial \psi }} \right)^{2} \hfill \\ - F\left( {\psi ;S} \right)\frac{{\partial^{2} F\left( {\psi ;S} \right)}}{{\partial \psi^{2} }} \hfill \\ \end{gathered} \right), \hfill \\ \end{gathered} $$

22 $$ \begin{gathered} \aleph_{G} \left[ {G\left( {\psi ;S} \right),F\left( {\psi ;S} \right)} \right] = \left( {1 + \frac{K}{2}} \right)\frac{{\partial^{2} G\left( {\psi ;S} \right)}}{{\partial \psi^{2} }} - K\left( {2G\left( {\psi ;S} \right) + \frac{{\partial^{2} F\left( {\psi ;S} \right)}}{{\partial \psi^{2} }}} \right) \hfill \\ - \left( {1 - \Phi_{1} } \right)^{2.5} \left( {1 - \Phi_{2} } \right)^{2.5} \left[ {\left( {1 - \Phi_{2} } \right)\left\{ {1 - \Phi_{1} + \Phi_{1} \frac{{\rho_{S1} }}{{\rho_{f} }}} \right\} + \Phi_{2} \frac{{\rho_{S2} }}{{\rho_{f} }}} \right] \hfill \\ \left( {\frac{{\partial F\left( {\psi ;S} \right)}}{\partial \psi }G\left( {\psi ;S} \right) - F\left( {\psi ;S} \right)\frac{{\partial F\left( {\psi ;S} \right)}}{\partial \psi }} \right), \hfill \\ \end{gathered} $$

23 $$ \begin{gathered} \aleph_{T} \left[ {T\left( {\psi ;S} \right),F\left( {\psi ;S} \right),G\left( {\psi ;S} \right)} \right] = \frac{{k_{hnf} }}{{k_{f} }}\frac{{\partial^{2} T\left( {\psi ;S} \right)}}{{\partial \psi^{2} }} + \left( {1 + \frac{K}{2}} \right)\frac{Ec\Pr }{{\left( {1 - \Phi_{1} } \right)^{2.5} \left( {1 - \Phi_{2} } \right)^{2.5} }} \hfill \\ \left( {\frac{{\partial^{2} F\left( {\psi ;S} \right)}}{{\partial \psi^{2} }}} \right)^{2} + \frac{K}{2}\frac{Ec\Pr }{{\left( {1 - \Phi_{1} } \right)^{2.5} \left( {1 - \Phi_{2} } \right)^{2.5} }}\left( \begin{gathered} \left( {\frac{{\partial^{2} F\left( {\psi ;S} \right)}}{{\partial \psi^{2} }}} \right)^{2} + 4\left( {G\left( {\psi ;S} \right)} \right)^{2} \hfill \\ - 4G\left( {\psi ;S} \right)\frac{{\partial^{2} F\left( {\psi ;S} \right)}}{{\partial \psi^{2} }} \hfill \\ \end{gathered} \right) \hfill \\ + CEc\Pr {\text{Re}} \left( {\frac{{\partial G\left( {\psi ;S} \right)}}{\partial \psi }} \right)^{2} + \Pr \left[ {\left( {1 - \Phi_{2} } \right)\left\{ {1 - \Phi_{1} + \Phi_{1} \frac{{\left( {\rho c_{p} } \right)_{S1} }}{{\left( {\rho c_{p} } \right)_{f} }}} \right\} + \Phi_{2} \frac{{\left( {\rho c_{p} } \right)_{S2} }}{{\left( {\rho c_{p} } \right)_{f} }}} \right] \hfill \\ \left( {F\left( {\psi ;S} \right)\frac{{\partial T\left( {\psi ;S} \right)}}{\partial \psi }} \right.\left. { - \frac{{\partial F\left( {\psi ;S} \right)}}{\partial \psi }T\left( {\psi ;S} \right)} \right) - \kappa \left( {F\left( {\psi ;S} \right)\frac{{\partial F\left( {\psi ;S} \right)}}{\partial \psi }\frac{{\partial T\left( {\psi ;S} \right)}}{\partial \psi }} \right. \hfill \\ \left. { + \left( {F\left( {\psi ;S} \right)} \right)^{2} \frac{{\partial^{2} T\left( {\psi ;S} \right)}}{{\partial \psi^{2} }}} \right) + \frac{{\sigma_{hnf} }}{{\sigma_{f} }}Ha\Pr Ec\left( {\frac{{\partial F\left( {\psi ;S} \right)}}{\partial \psi }} \right)^{2} , \hfill \\ \end{gathered} $$

*k*th-*order problems*

*k*th-order problems are

24 $$ L_{F} \left[ {F_{k} \left( \psi \right) - \chi_{k} F_{k - 1} \left( \psi \right)} \right] = \hbar_{k} \Re_{k}^{F} \left( \psi \right), $$

25 $$ L_{G} \left[ {G_{k} \left( \psi \right) - \chi_{k} G_{k - 1} \left( \psi \right)} \right] = \hbar_{k} \Re_{k}^{G} \left( \psi \right), $$

26 $$ L_{T} \left[ {T_{k} \left( \psi \right) - \chi_{k} T_{k - 1} \left( \psi \right)} \right] = \hbar_{T} \Re_{k}^{T} \left( \psi \right), $$

27 $$ F_{k} \left( 0 \right) = F^{\prime}_{k} \left( 0 \right) = G^{\prime}_{k} \left( 0 \right) = \,T_{k} \left( 0 \right)\, = F^{\prime}_{k} \left( \infty \right) = \,G^{\prime}_{k} \left( \infty \right) = T_{k} \left( \infty \right) = 0. $$

28 $$ \begin{gathered} \Re_{k}^{F} \left( \psi \right) = \left( {1 + K} \right)F^{\prime\prime\prime}_{k - 1} + KG^{\prime}_{k - 1} + \frac{{\sigma_{hnf} }}{{\sigma_{f} \left[ {\left( {1 - \Phi_{2} } \right)\left\{ {1 - \Phi_{1} + \Phi_{1} \frac{{\rho_{S1} }}{{\rho_{f} }}} \right\} + \Phi_{2} \frac{{\rho_{S2} }}{{\rho_{f} }}} \right]}}HaF^{\prime}_{k - 1} \hfill \\ - \left( {1 - \Phi_{1} } \right)^{2.5} \left( {1 - \Phi_{2} } \right)^{2.5} \left[ {\left( {1 - \Phi_{2} } \right)\left\{ {1 - \Phi_{1} + \Phi_{1} \frac{{\rho_{S1} }}{{\rho_{f} }}} \right\} + \Phi_{2} \frac{{\rho_{S2} }}{{\rho_{f} }}} \right]\left( {F_{k - 1}^{{{\prime }2}} - \sum\limits_{j = 0}^{k - 1} {\left( {F_{k - 1 - j} F^{\prime\prime}_{k - 1} } \right)} } \right), \hfill \\ \end{gathered} $$

29 $$ \begin{gathered} \Re_{k}^{G} \left( \psi \right) = \left( {1 + \frac{K}{2}} \right)G^{\prime\prime}_{k - 1} - K\left( {2G_{k - 1} + F^{\prime\prime}_{k - 1} } \right) \hfill \\ -  \left( {1 - \Phi_{1} } \right)^{2.5} \left( {1 - \Phi_{2} } \right)^{2.5} \left[ {\left( {1 - \Phi_{2} } \right)\left\{ {1 - \Phi_{1} + \Phi_{1} \frac{{\rho_{S1} }}{{\rho_{f} }}} \right\} + \Phi_{2} \frac{{\rho_{S2} }}{{\rho_{f} }}} \right]\left( {\sum\limits_{j = 0}^{k - 1} {\left( {F^{\prime}_{k - 1 - j} G_{k - 1} } \right)} - \sum\limits_{j = 0}^{k - 1} {\left( {F_{k - 1 - j} G^{\prime}_{k - 1} } \right)} } \right), \hfill \\ \end{gathered} $$

30 $$ \begin{gathered} \Re_{k}^{T} \left( \psi \right) = \frac{{k_{hnf} }}{{k_{f} }}T^{\prime\prime}_{k - 1} + \frac{{\sigma_{hnf} }}{{\sigma_{f} }}Ha\Pr EcF_{k - 1}^{{{\prime }2}} + \left( {1 + \frac{K}{2}} \right)\frac{Ec\Pr }{{\left( {1 - \Phi_{1} } \right)^{2.5} \left( {1 - \Phi_{2} } \right)^{2.5} }}F_{k - 1}^{{{\prime \prime }2}} \hfill \\ + \frac{K}{2}\frac{Ec\Pr }{{\left( {1 - \Phi_{1} } \right)^{2.5} \left( {1 - \Phi_{2} } \right)^{2.5} }}\left( {F_{k - 1}^{{{\prime \prime }2}} + 4G_{k - 1}^{2} - 4\sum\limits_{j = 0}^{k - 1} {\left( {G_{k - 1 - j} F^{\prime\prime}_{k - 1} } \right)} } \right) + CEc\Pr {\text{Re}} G_{k - 1}^{{{\prime }2}} \hfill \\ + \Pr \left[ {\left( {1 - \Phi_{2} } \right)\left\{ {1 - \Phi_{1} + \Phi_{1} \frac{{\left( {\rho c_{p} } \right)_{S1} }}{{\left( {\rho c_{p} } \right)_{f} }}} \right\} + \Phi_{2} \frac{{\left( {\rho c_{p} } \right)_{S2} }}{{\left( {\rho c_{p} } \right)_{f} }}} \right]\left( {\sum\limits_{j = 0}^{k - 1} {\left( {F_{k - 1 - j} T^{\prime}_{k - 1} } \right)} - \sum\limits_{j = 0}^{k - 1} {\left( {F^{\prime}_{k - 1 - j} T_{k - 1} } \right)} } \right) \hfill \\ - \kappa \left( {\sum\limits_{j = 0}^{k - 1} {\left( {F_{k - 1 - j} F^{\prime}_{k - 1} T^{\prime}_{k} } \right)} + \sum\limits_{j = 0}^{k - 1} {\left( {F_{k - 1 - j}^{2} T^{\prime\prime}_{k - 1} } \right)} } \right). \hfill \\ \end{gathered} $$

When $S = 0$ and $S = 1$, we can write

31 $$ F\left( {\psi ;0} \right) = F_{0} \left( \psi \right),\,\,\,\,F\left( {\psi ;1} \right) = F\left( \psi \right), $$

32 $$ G\left( {\psi ;0} \right) = G_{0} \left( \psi \right),\,\,\,\,G\left( {\psi ;1} \right) = G\left( \psi \right), $$

33 $$ T\left( {\psi ;0} \right) = T_{0} \left( \psi \right),\,\,\,\,T\left( {\psi ;1} \right) = T\left( \psi \right), $$

when $S$ fluctuates from 0 to 1, the results varies from initial to final solutions. By Taylor’s series expansion

34 $$ F\left( {\psi ;S} \right) = F_{0} \left( \psi \right) + \sum\limits_{k = 1}^{\infty } {F_{k} \left( \psi \right)} S^{k} ,\,\,\,\left. {F_{k} = \frac{1}{k!}\frac{{\partial^{k} F\left( {\psi ;S} \right)}}{{\partial S^{k} }}} \right|_{S = 0} , $$

35 $$ G\left( {\psi ;S} \right) = G_{0} \left( \psi \right) + \sum\limits_{k = 1}^{\infty } {G_{k} \left( \psi \right)} S^{k} ,\,\,\,\left. {G_{k} = \frac{1}{k!}\frac{{\partial^{k} G\left( {\psi ;S} \right)}}{{\partial S^{k} }}} \right|_{S = 0} , $$

36 $$ T\left( {\psi ;S} \right) = T_{0} \left( \psi \right) + \sum\limits_{k = 1}^{\infty } {T_{k} \left( \psi \right)} S^{k} ,\,\,\,\left. {T_{k} = \frac{1}{k!}\frac{{\partial^{k} T\left( {\psi ;S} \right)}}{{\partial S^{k} }}} \right|_{S = 0} . $$

The series (34) and (36) converge at $S = 1$, that is

37 $$ F\left( \psi \right) = F_{0} \left( \psi \right) + \sum\limits_{k = 1}^{\infty } {F_{k} \left( \psi \right)} , $$

38 $$ G\left( \psi \right) = G_{0} \left( \psi \right) + \sum\limits_{k = 1}^{\infty } {G_{k} \left( \psi \right)} , $$

39 $$ T\left( \psi \right) = T_{0} \left( \psi \right) + \sum\limits_{k = 1}^{\infty } {T_{k} \left( \psi \right)} , $$

where

40 $$ \chi_{k} = \left\{ \begin{gathered} 0,\,\,\,\,\,k \le 1, \hfill \\ 1,\,\,\,\,\,\,k > 1. \hfill \\ \end{gathered} \right. $$

## Convergence solution

**Figure 2:**
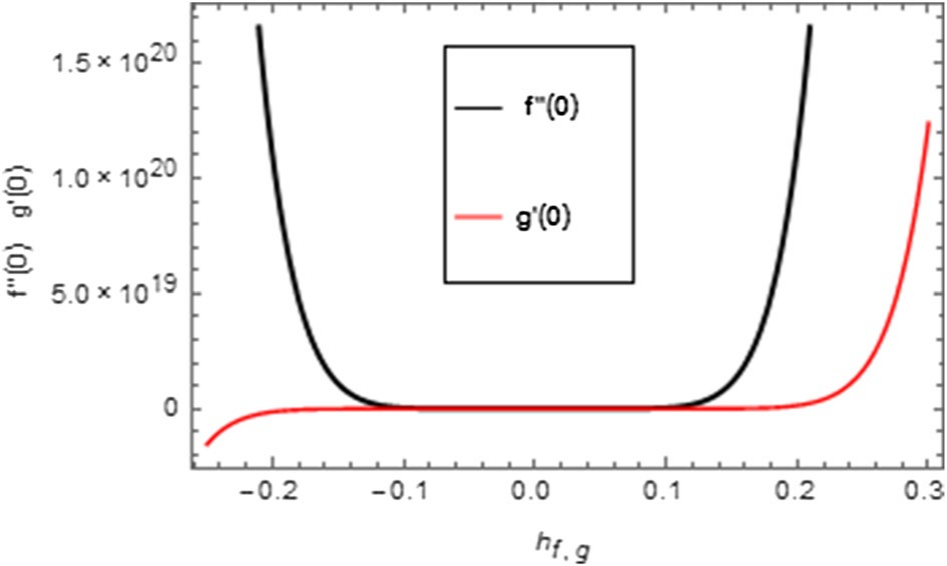
$\hbar$-curves for velocity field and micro-rotation field.

**Figure 3:**
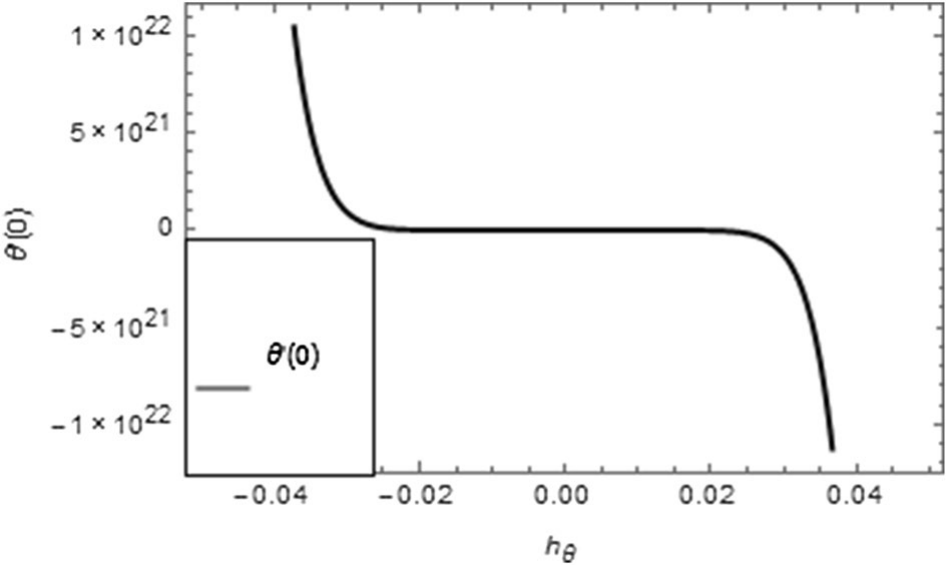
$\hbar$-curve for temperature field.

The supplementary factors $\hbar_{f} ,\,\,\hbar_{g}$ and $\hbar_{\theta }$ convoluted in the series solutions for velocity, micro-rotation, and temperature fields of hybrid nanofluid are characterized as convergence regulator factors. These factors are used to stabilize the convergence of the demonstrated problem. The suitable standards of the $\hbar_{f} ,\,\,\hbar_{g}$ and $\hbar_{\theta }$ are $- 0.15 \le \hbar_{f} \le 0.15$, $- 0.2 \le \hbar_{g} \le 0.22$ and $- 0.03 \le \hbar_{\theta } \le 0.03$ which are shown in Figs. 2 and 3.

## Analytical and numerical comparison

**Figure 4:**
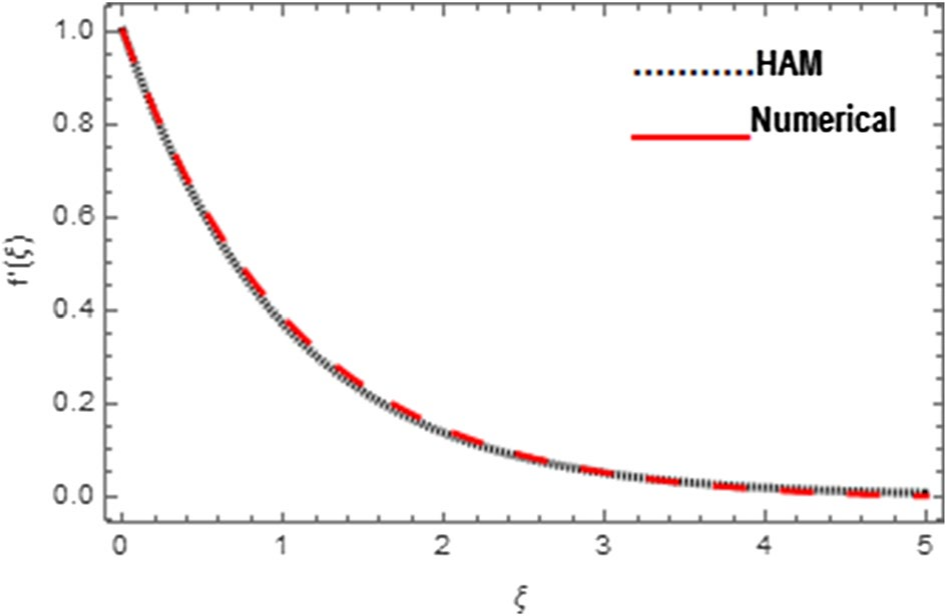
Association between HAM and Shooting techniques for velocity field.

**Figure 5:**
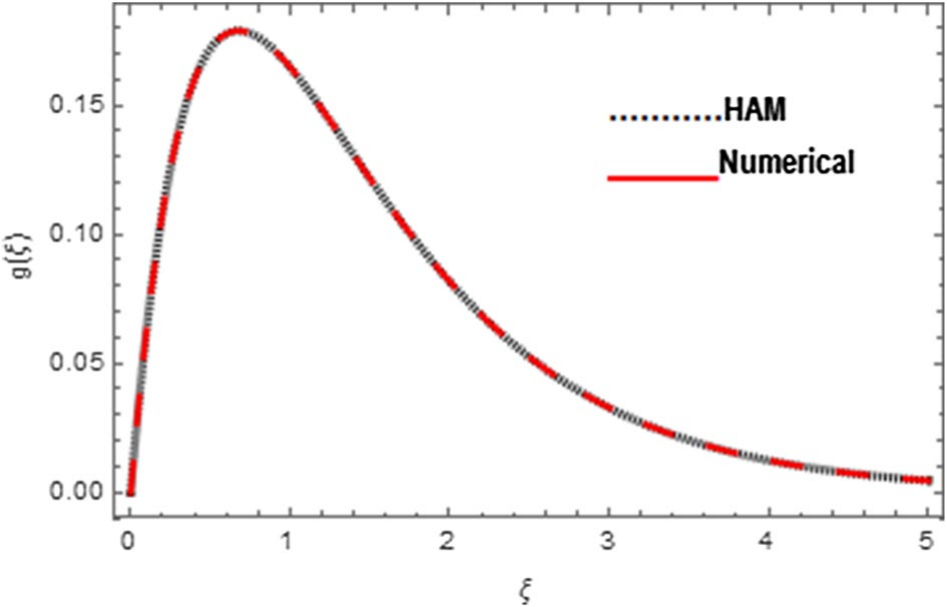
Association between HAM and Shooting techniques for microrotation field.

**Figure 6:**
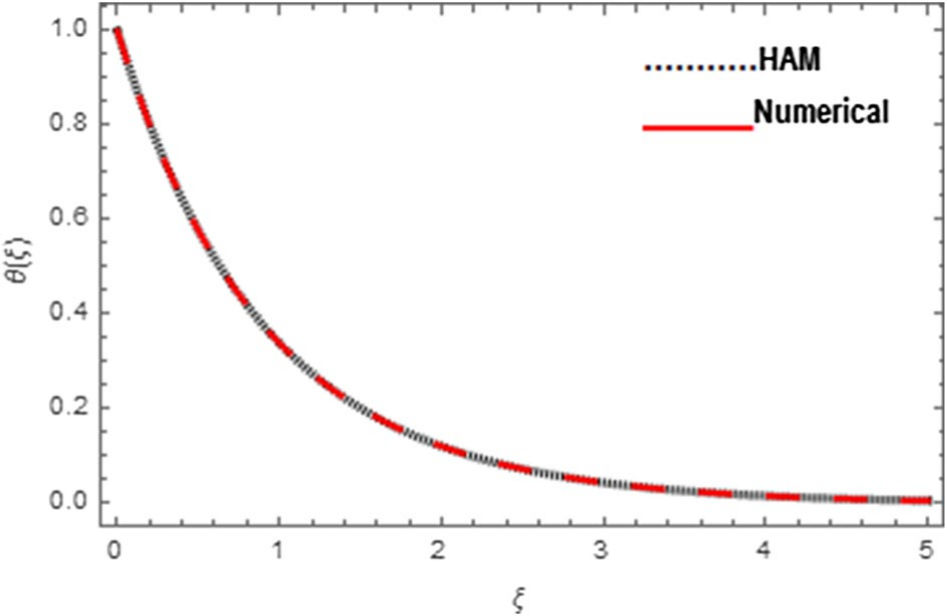
Association between HAM and Shooting techniques for temperature field.

Association between analytical and numerical procedures for velocity, micro-rotation and thermal fields are presented here. Both the procedures are preserved with the conventional computer programs and authenticated by reproducing the consequences to the manageable standard and have totaled with mathematica software. Figure 4 specifies the assessment of HAM and shooting methods for velocity field $f^{\prime}\left( \xi \right)$. Similarly Figs. 5 and 6 denote the assessment of HAM and shooting methods for micro-rotation field $g\left( \xi \right)$ and thermal field $\theta \left( \xi \right)$, respectively. Also Table 2 is displayed for the assessment of HAM and shooting procedures.

**Table 2:** Numerical association between HAM and Shooting techniques for velocity, micro-rotation, and temperature fields.

$\xi$	Velocity field		Micro-rotation field		Temperature field	
	HAM	Shooting	HAM	Shooting	HAM	Shooting
0.0	− 7.5 × 10^–19^	0.000000	− 3.53 × 10^–9^	0.000000	− 3.97 × 10^–9^	0.000000
0.5	0.393940	0.397855	0.523062	0.514206	0.599470	0.599799
1.0	0.633330	0.644735	0.372430	0.364476	0.361400	0.361695
1.5	0.778692	0.797385	0.246020	0.239908	0.218506	0.218717
2.0	0.866920	0.891134	0.156424	0.152343	0.132305	0.132444
2.5	0.920454	0.948022	0.097504	0.094893	0.080171	0.080258
3.0	0.952933	0.981823	0.060101	0.058468	0.048600	0.048654
3.5	0.972663	1.001170	0.036806	0.035797	0.029468	0.029501
4.0	0.984587	1.011490	0.022453	0.021835	0.017870	0.017890
4.5	0.991836	1.016185	0.013666	0.013289	0.010837	0.010849
5.0	0.996233	1.017429	0.008306	0.008076	0.006572	0.006580

## Results and discussion

**Figure 7:**
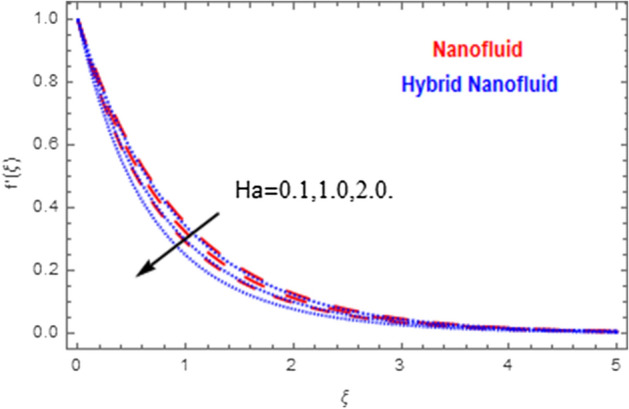
Variation in velocity field of nanofluid (${\text{Al}}_{2} {\text{O}}_{3}$) and hybrid nanofluid (${\text{Al}}_{2} {\text{O}}_{3} ,\,{\text{Cu}}$) against different estimations of $Ha$.

**Figure 8:**
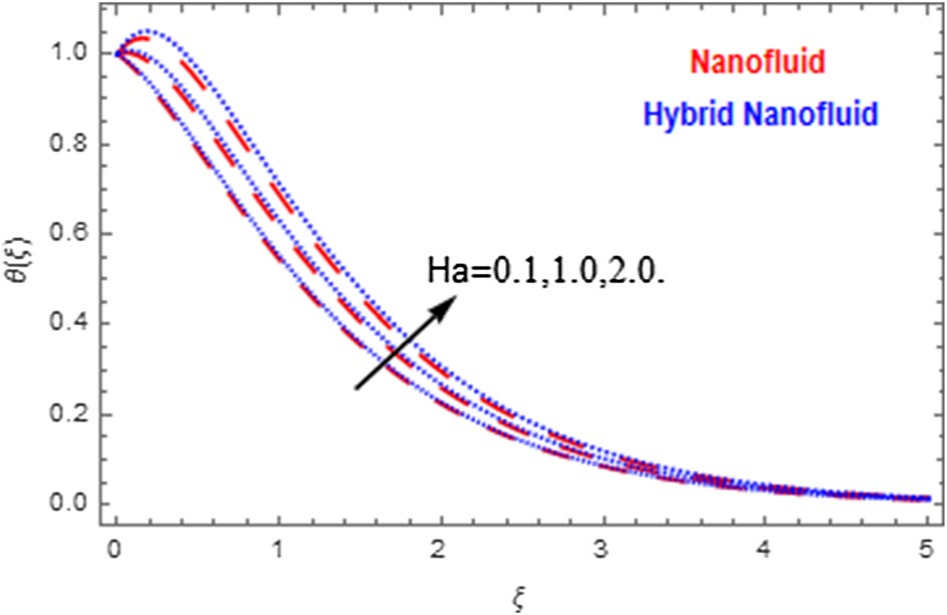
Variation in temperature field of nanofluid (${\text{Al}}_{2} {\text{O}}_{3}$) and hybrid nanofluid (${\text{Al}}_{2} {\text{O}}_{3} ,\,{\text{Cu}}$) against different estimations of $Ha$.

**Figure 9:**
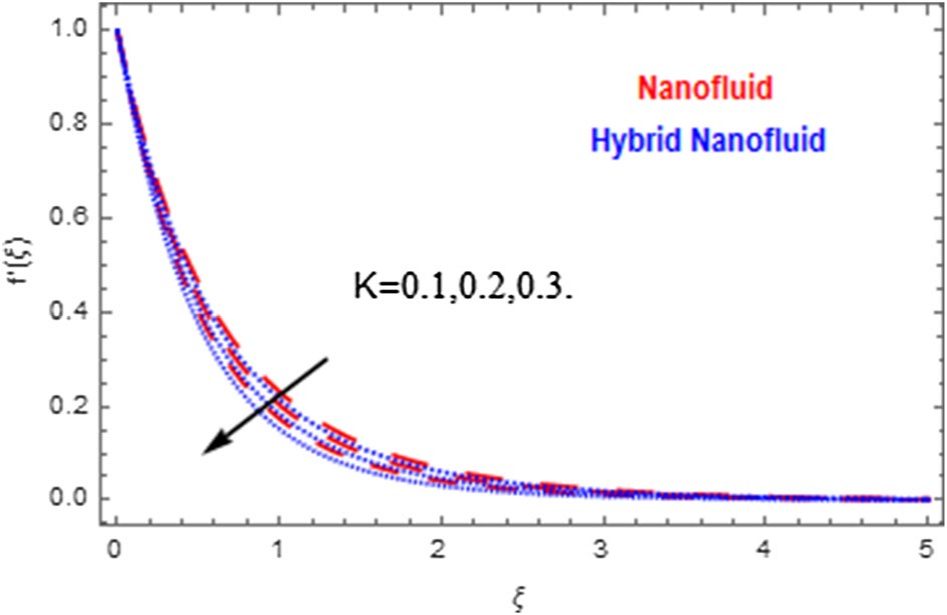
Variation in velocity field of nanofluid (${\text{Al}}_{2} {\text{O}}_{3}$) and hybrid nanofluid (${\text{Al}}_{2} {\text{O}}_{3} ,\,{\text{Cu}}$) against different estimations of $K$.

**Figure 10:**
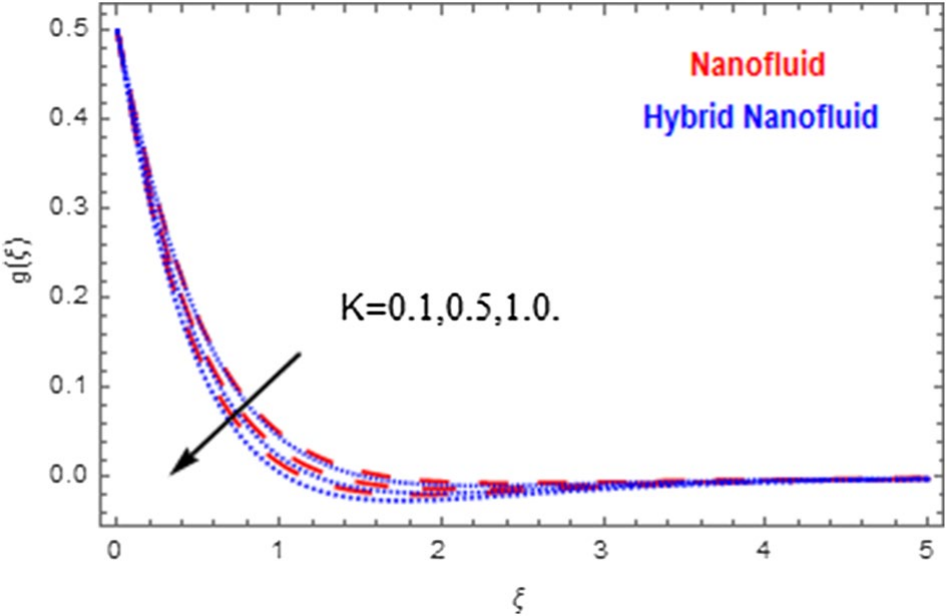
Variation in microrotation field of nanofluid (${\text{Al}}_{2} {\text{O}}_{3}$) and hybrid nanofluid (${\text{Al}}_{2} {\text{O}}_{3} ,\,{\text{Cu}}$) against different estimations of $K$ when $n = 0.5$.

**Figure 11:**
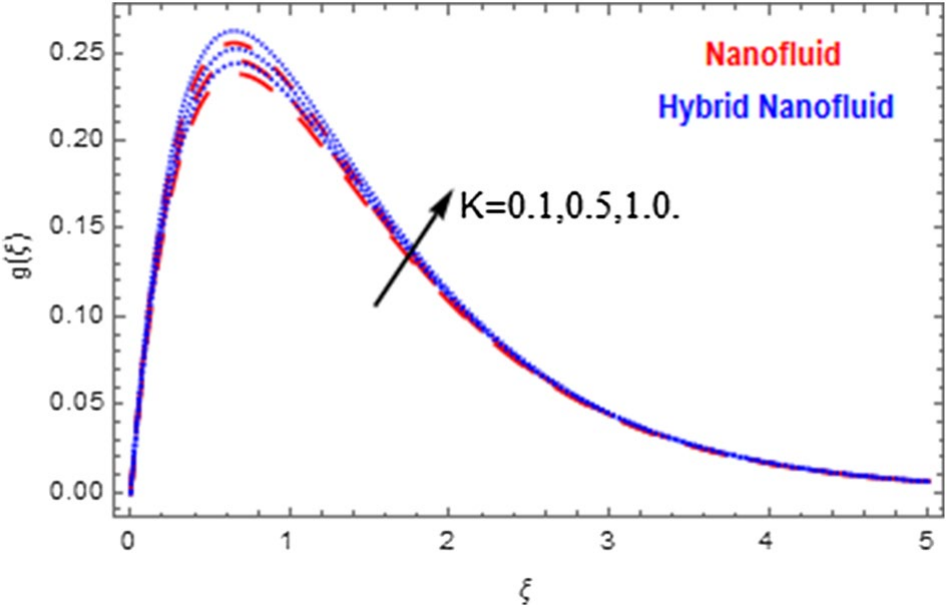
Variation in microrotation field of nanofluid (${\text{Al}}_{2} {\text{O}}_{3}$) and hybrid nanofluid (${\text{Al}}_{2} {\text{O}}_{3} ,\,{\text{Cu}}$) against different estimations of $K$ when $n = 0$.

**Figure 12:**
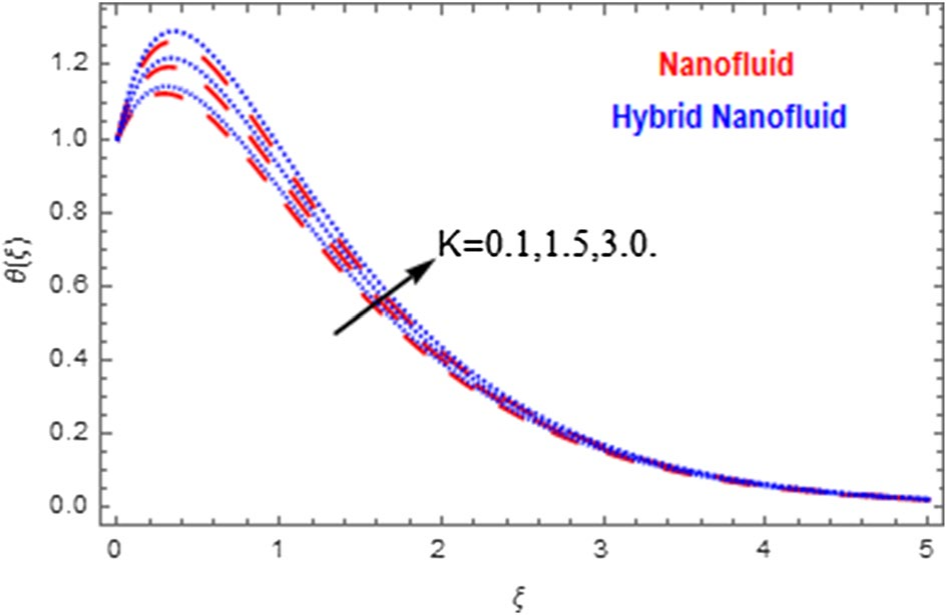
Variation in temperature field of nanofluid (${\text{Al}}_{2} {\text{O}}_{3}$) and hybrid nanofluid (${\text{Al}}_{2} {\text{O}}_{3} ,\,{\text{Cu}}$) against different estimations of $K$.

**Figure 13:**
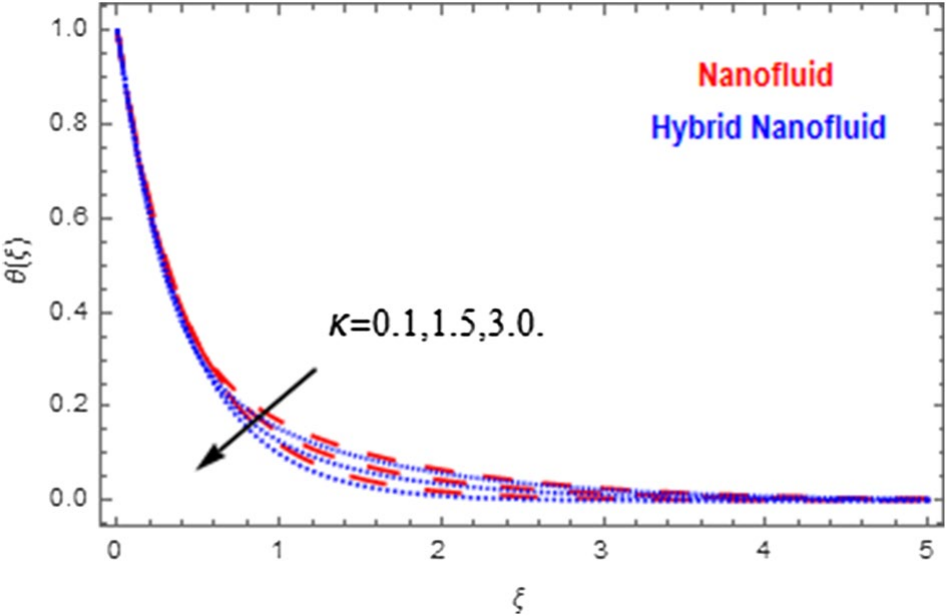
Variation in temperature field of nanofluid (${\text{Al}}_{2} {\text{O}}_{3}$) and hybrid nanofluid (${\text{Al}}_{2} {\text{O}}_{3} ,\,{\text{Cu}}$) against different estimations of $\kappa$.

**Figure 14:**
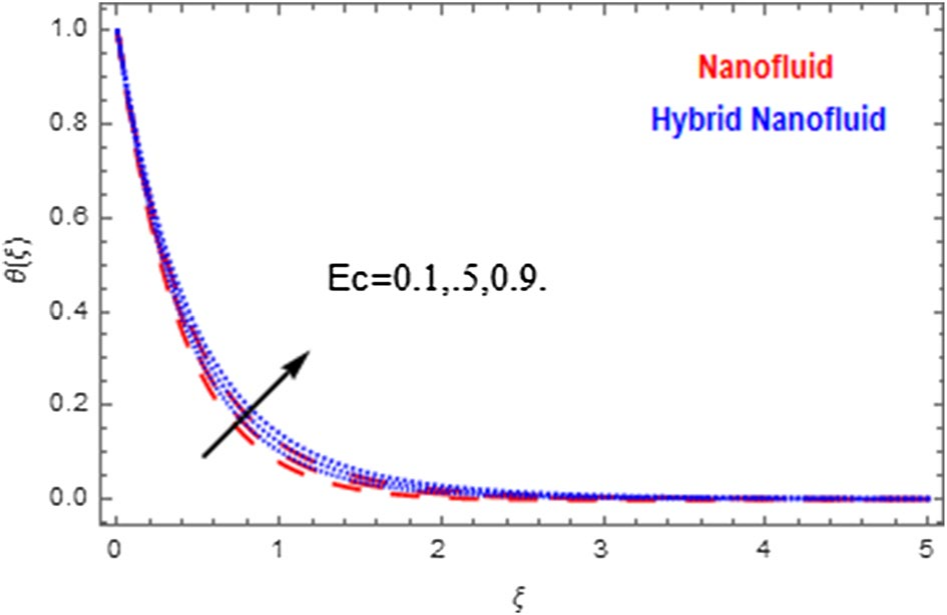
Variation in temperature field of nanofluid (${\text{Al}}_{2} {\text{O}}_{3}$) and hybrid nanofluid (${\text{Al}}_{2} {\text{O}}_{3} ,\,{\text{Cu}}$) against different estimations of $Ec$.

**Figure 15:**
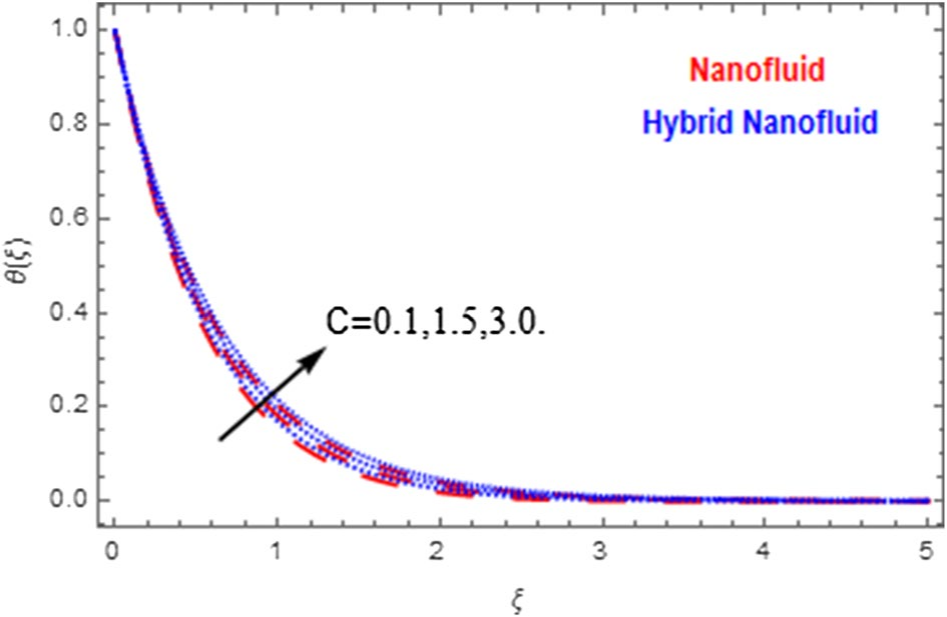
Variation in temperature field of nanofluid ($${\text{Al}}_{2} {\text{O}}_{3}$$) and hybrid nanofluid ($${\text{Al}}_{2} {\text{O}}_{3} ,\,{\text{Cu}}$$$${\text{Al}}_{2} {\text{O}}_{3} ,\,{\text{Cu}}$$) against different estimations of $$C$$.

**Figure 16:**
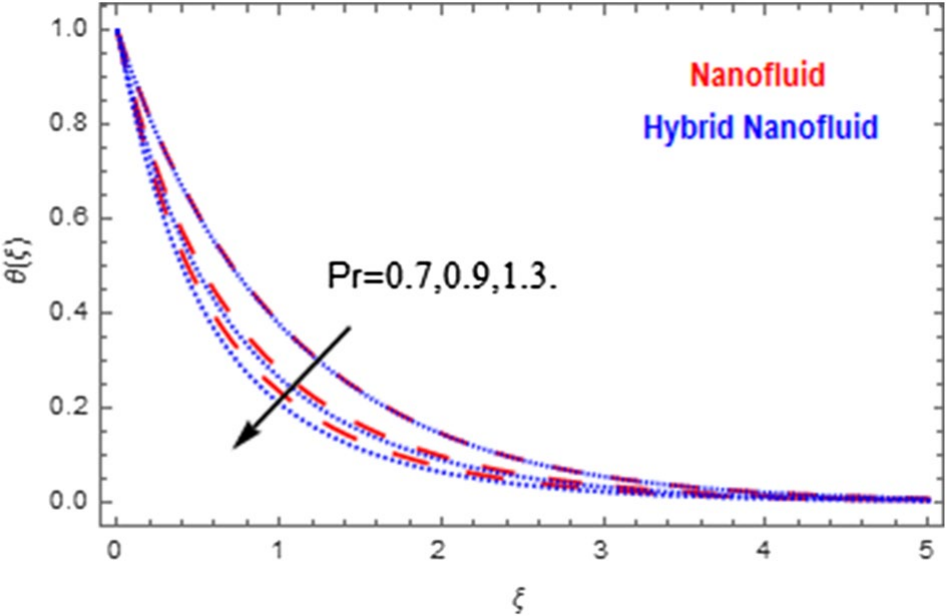
Variation in temperature field of nanofluid (${\text{Al}}_{2} {\text{O}}_{3}$) and hybrid nanofluid (${\text{Al}}_{2} {\text{O}}_{3} ,\,{\text{Cu}}$) against different estimations of $\Pr$.

This segment deals with the physical impact of constraints on the nanofluid (${\text{Al}}_{2} {\text{O}}_{3}$) and hybrid nanofluid (${\text{Al}}_{2} {\text{O}}_{3} ,\,{\text{Cu}}$) flows when, $\Pr = 2.3$, $Ha = 1.5$, $Ec = 0.5$, $\kappa = 1.0$, $K = 0.6$, and $C = 0.3$. These impacts are displayed in Figs. 7, 8, 9, 10, 11, 12, 13, 14, 15, 16. The deviation in velocity field contrasted with Hartmann number is depicted in Fig. 7. As the estimations of Hartmann number intensifies, the boundary layer thickness of the velocity field escalates. This is attributable to the datum that the existence of a Hartmann number is measured by the Lorentz force, so throughout a delaying force is conceived throughout the velocity field. Thus as the estimations of $Ha$ grows, so is the restricting force and thus the velocity field declines. The contrary impression of Hartmann number on temperature field is observed in Fig. 8. It is found that as the estimations of $Ha$ increase, the temperature at the surface and the thermal boundary layer thickness declines. The variation in velocity field against micropolar fluid parameter is displayed in Fig. 9. It is remarkable to know that $n = 0$ is the situation when the motion of the nanofluid and hybrid nanoparticles disappears at the surface of the body. It is depicted here that higher estimations of micropolar fluid parameter declines the velocity field of the flow. It is furthermore important and well-known here that the boundary layer thickness is smaller for the hybrid nanofluid than that of nanofluid. Figures 10 and 11 exhibit the variation in micro-rotation field against micropolar fluid parameter for the situations when $n = 0.5$ and $n = 0$, respectively. For the case when $n = 0.5$, the micro-rotation field declines with the higher estimations of micropolar fluid parameter. This deviation is publicized in Fig. 10. For the case when $n = 0$, the micro-rotation field exhibits the diverse impression with escalation in micropolar fluid parameter. This effect is shown in Fig. 11. An increasing behavior in thermal field with the escalating estimations of micropolar fluid parameter is displayed in Fig. 12. Physically, the rise in micropolar fluid parameter enables heightening in thermal boundary layer thickness, and thus the thermal field enhances. The relation of the thermal field versus relaxation time parameter $\kappa$ is depicted in Fig. 13. The temperature relaxation time factor decreases the thermal profile and the related thermal boundary layer thickness. Actually, this demonstrates that the gradual existence of $\kappa$ takes additional time to change the energy from tightly loaded fluid particles to lower active flow particles. It demonstrates a representation of the features of non-conductive fluid material. The Influence of Eckert number on thermal field of the nanofluid and hybrid nanofluid is put on a display in Fig. 14. The heightening Eckert number heightens the thermal field. In the case of a large magnetic force system, the major disruption of the temperature field induced by each member is an interesting result. Such physical phenomena are attributable to the cumulative effect heat energy stored in the nanofluid and hybrid nanofluid due to fractional heating. The variation in thermal field against material parameter is displayed in Fig. 15. An increasing impact is seen here. The higher estimations of material parameter raise the thermal field of the nanofuid and hybrid nanofluid. The variation in thermal field of the nanofluid and hybrid nanofluid versus Prandtl number $\Pr$ is displayed in Fig. 16. The graph shows that thermal field and thermal layer thickness decline whenever the $\Pr$ values boost. This really is caused by the fact that the greater number of Prandtl, fluids will have a comparatively low conductivity, that mostly diminishes the heat transfer and the thickness of the thermal fluid flow and therefore the temperature of the fluid reduces. Growing $\Pr$ is an improvement in the thermal transfer rate on the surface, even though the thermal gradient as on the surface grows. The effect of $\Pr$ on Newtonian fluids is close to what we're seeing in nanofluid. These characteristics are thus already retained from nanofluids.

Table 3 displays the variation in $C_{f}$, $C_{g}$ and $Nu$ for nanofluid (${\text{Al}}_{2} {\text{O}}_{3}$) and hybrid nanofluids (${\text{Al}}_{2} {\text{O}}_{3} ,\,\,{\text{Cu}}$) against different estimations of $K$ when $\Pr = 2.36,\,\,{\text{Re}} = 15,\,\,Ha = 0.3,\,\,\kappa = 0.5$ and $Ec = 0.001$. The first portion is shown for the variation in $C_{f}$, $C_{g}$ and $Nu$ when $n = 0.5$. The escalating estimations of $K$ heighten $C_{f}$ and $C_{g}$ while reduce $Nu$ for both nanofluid and hybrid nanofluid. The second portion is shown for the variation in $C_{f}$, $C_{g}$ and $Nu$ when $n = 0.0$. The escalating estimations of $K$ heightens $C_{f}$ and $C_{g}$ while reduce $Nu$ for both nanofluid and hybrid nanofluid.

**Table 3:** Variation in $C_{f}$, $C_{g}$ and $Nu$ for nanofluid (${\text{Al}}_{2} {\text{O}}_{3}$) and hybrid nanofluids (${\text{Al}}_{2} {\text{O}}_{3} ,\,\,{\text{Cu}}$) against different values of $K$ when $\Pr = 2.36,\,\,{\text{Re}} = 15,\,\,Ha = 0.3,\,\,\kappa = 0.5$ and $Ec = 0.001$.

Nanofluid				Hybrid nanofluid		
$$K$$	$$C_{f}$$	$$C_{g}$$	$$Nu$$	$$C_{f}$$	$$C_{g}$$$$C_{g}$$	$$Nu$$
	$n = 0.5$			$n = 0.5$		
0.0	1.116551	4.984882	1.454353	0.845883	3.776366	1.632642
0.8	2.427641	9.040594	1.253680	1.682611	6.383817	1.425742
1.5	3.364413	12.343846	1.158973	2.283628	8.560773	1.379473
2.0	3.947586	14.656566	1.126438	2.661480	10.092277	1.356843

$K$	$C_{f}$	$C_{g}$	$Nu$	$C_{f}$	$C_{g}$	$Nu$
	$n = 0.0$			$n = 0.0$		
0.5	1.896726	0.305409	1.736808	1.375964	0.206398	1.986432
1.0	2.906938	0.613401	1.646785	2.037898	0.410905	1.824643
1.5	4.037389	0.932532	1.569084	2.781046	0.622338	1.710583
2.0	5.230850	1.230850	1.497533	3.568901	0.837481	1.634643

## Conclusion

The analytical and numerical analysis on the effect of hybrid nanoparticles (${\text{Cu}}$ and ${\text{Al}}_{2} {\text{O}}_{3}$) on the thermal efficiency of nano-structured nanoparticles (micropolar fluid) with Cattaneo-Christov heat flux model is discussed here. The comparison of both approaches is deliberated here as well. The main findings are as follows:

1. Continuous dispersion of ${\text{Cu}}$ and ${\text{Al}}_{2} {\text{O}}_{3}$ leads in an optimal increase in thermal efficiency compared to single-type dispersion of nanoparticles, say ${\text{Cu}}$.
2. A continuous reduction in macro-velocity field is observed against increasing micropolar parameter and Hartmann number.
3. An opposite effect in micro-velocity field against micropolar parameter when $n = 0.5$ and $n = 0$ is observed.
4. An escalating conduct in thermal field is observed against the increasing estimations of Hartmann number, micropolar parameter, Eckert number, and material parameter while Prandtl number and thermal relaxation parameter has opposite conduct on thermal field.
5. Both the HAM and Shooting approaches are agreed with presented model and have shown quite closed comparison.

## Abbreviations

$$A,\,d,\,a$$: Consistency parameters
$$B_{0}$$: Magnetic induction
$$c_{p}$$: Specific heat
$$C$$: Material parameter
$$Ec$$: Eckert number
$$Ha$$: Hartmann number
$$j$$: Length scale
$$k$$: Thermal conductivity
$$K$$: Micropolar parameter
$$n$$: Constant
$$\Pr$$: Prandtl number
$$T$$: Temperature
$$T_{w}$$: Surface temperature
$T_{\infty }$: Temperature outside from the surface
$$u,\,v$$: Velocity components
$$\alpha ,\,\beta ,\,\gamma$$: Material parameters
$$\Phi_{1} ,\,\Phi_{2}$$: Volume frictions
$$\psi$$: Stream function
$$\sigma$$: Electrical conductivity
$$\rho$$: Density
$\gamma$: Spin gradient viscosity
$\kappa$: Thermal relaxation time

## Subscripts

$$f$$: Fluid
$$nf$$: Nanofluid
$$hnf$$: Hybrid nanofluid
